# Melanoma in Northwestern Romania: An Analysis of Epidemiological and Histopathological Characteristics and Associated Risk Factors

**DOI:** 10.3390/jcm14030946

**Published:** 2025-02-01

**Authors:** Adina Patricia Apostu, Loredana Ungureanu, Andra Piciu, Ștefan Cristian Vesa, Salomea Ruth Halmagyi, Ioana Irina Trufin, Simona Frațilă, Gabriela Iancu, Simona Corina Șenilă

**Affiliations:** 1 Department of Dermatology, “Iuliu Hațieganu” University of Medicine and Pharmacy, 400012 Cluj-Napoca, Romania; adinna.apostu@yahoo.com (A.P.A.);; 2 Clinical Hospital of Infectious Diseases, 400000 Cluj-Napoca, Romania; 3 Department of Dermatology, Emergency County Hospital, 400006 Cluj-Napoca, Romania; 4 Department of Oncology, ”Iuliu Hațieganu” University of Medicine and Pharmacy, 400012 Cluj-Napoca, Romania; 5 Department of Pharmacology, Toxicology and Clinical Pharmacology, “Iuliu Hațieganu” University of Medicine and Pharmacy, 400012 Cluj-Napoca, Romania; 6 Faculty of Medicine and Pharmacy, University of Oradea, 410073 Oradea, Romania; sfratila@uoradea.ro; 7 Clinical Emergency County Hospital, 410167 Oradea, Romania; 8 Department of Dermatology, Faculty of Medicine “Lucian Blaga” University of Sibiu, 550024 Sibiu, Romania; gabriela.iancu@ulbsibiu.ro; 9 Clinic of Dermatology, County Emergency Hospital Sibiu, 550012 Sibiu, Romania

**Keywords:** melanoma, risk factors, epidemiology

## Abstract

**Background**: Cutaneous melanoma (CM) is a malignant tumor originating from melanocytes. Despite improvements in prevention, Central and Eastern European countries continue to report higher rates of advanced-stage melanoma and lower survival rates. This study aims to characterize CM and the associated risk factors in Northwestern Romania. **Methods**: This cross-sectional cohort study was conducted in Cluj and Bihor counties. Between January 2023 and May 2024, 172 patients with histopathologically confirmed melanoma completed a standardized questionnaire addressing demographics, sun exposure history, nevi count, and melanoma-specific characteristics. **Results**: The median age at diagnosis of participants was 44 years. The median Breslow index (BI) was 1.5 mm, and 39% of cases presented with a BI > 2 mm. Superficial spreading melanoma (SSM) was the most common subtype, predominantly affecting women, while nodular melanoma (NM) was more frequent in men. Higher BI was associated with NM and acral lentiginous melanoma (ALM). Limbs were women’s most frequent tumor site, whereas the trunk was predominant in men. Significant associations were observed between younger age at diagnosis and factors such as high nevus count, indoor activity, and smoking status. Rural residents reported a higher history of sunburns compared to urban residents. **Conclusions**: Our findings underscore the importance of targeted public health interventions to promote early detection and primary prevention of melanoma. Establishing a national melanoma registry is crucial to improving epidemiological surveillance and reducing the burden of melanoma in Romania.

## 1. Introduction

Cutaneous melanoma is a malignant tumor originating from melanocytes and has demonstrated rising incidence in European populations despite preventive efforts [1]. In Europe, melanoma incidence varies significantly by country. For example, Norway has the highest incidence (29.5 per 100,000), while Romania reports the lowest (3.4 per 100,000) [2]. Conversely, Central and Eastern European countries report higher frequencies of advanced-stage melanoma and lower survival rates [3]. For instance, approximately 25% of melanoma patients in Bulgaria and Romania present with stage III or IV disease, and less than 10% of cases are classified as thin melanomas [4,5]. Although melanoma accounts for only about 4% of all skin cancers, it is responsible for over 80% of skin cancer-related deaths. The prognosis is favorable if melanoma is detected early [1,6,7].

Melanoma is a multifactorial disease, and the complete array of risk factors is not yet fully understood. Major risk factors include UV exposure and a history of severe sunburns [8]. Other contributing factors are phenotypic characteristics (such as skin type), high nevus count, family history of melanoma, and the presence of dysplastic nevi [9,10]. Specific characteristics, such as blonde or red hair, light skin tone, light eye color, and poor tanning ability, are well-established predisposing factors for cutaneous melanoma [11]. Additionally, immunosuppression and indoor tanning are acknowledged as significant risk factors for melanoma [12].

Nevi, benign melanocytic tumors, are considered both precursors to and markers of elevated melanoma risk [13,14]. A positive family history of melanoma is an independent risk factor, with approximately 10% of melanoma patients reporting a familial history of the disease [15].

Melanoma patients face an elevated risk of developing additional melanomas. As survival rates improve, more patients live longer post-diagnosis, consequently increasing the likelihood of secondary melanoma development [16]. Studies indicate that the incidence of a second melanoma ranges from less than 1% to over 10% within five years, highlighting the critical need for effective secondary prevention strategies [16,17].

While global studies have examined melanoma epidemiology and risk factors, data from Eastern Europe remain limited, and only a few countries in the region maintain high-quality cancer registries [3,4,18,19,20]. Our study aims to provide a comprehensive analysis of the epidemiological and histopathological characteristics of melanoma in Northwestern Romania, with a focus on identifying demographic trends, clinical features, and risk factors such as sun exposure and nevus count. Additionally, the study seeks to explore the relationship between tumor thickness and patient characteristics, including age, sex, and tumor localization.

## 2. Materials and Methods

Study design: this observational, cross-sectional cohort study was conducted in Cluj and Bihor counties, located in the northwestern region of Romania.

Study population: Between 1 January 2023 and 31 May 2024, adult patients diagnosed with melanoma who attended annual follow-up visits at the Department of Dermatology, Emergency County Hospital Cluj, Prof. Dr. I. Chiricuta Institute of Oncology Cluj, and Emergency County Hospital Oradea were invited to participate. They completed a standardized paper-based questionnaire addressing epidemiological aspects and risk factors for melanoma. Written informed consent was obtained from all participants, and the study received ethical approval from the Iuliu Hațieganu University of Medicine and Pharmacy Ethics Committee (Cluj-Napoca, Romania) (no 296/28 November 2022).

Inclusion criteria were adults (≥18 years) with histopathologically confirmed invasive cutaneous melanoma attending regular follow-ups at the aforementioned hospitals and providing informed consent. Exclusion criteria encompassed patients without a confirmed melanoma diagnosis, those with extracutaneous or melanoma of unknown origin, patients with in situ melanoma, individuals under 18 years of age, patients who did not fully complete the questionnaire, and those who declined to provide informed consent.

Data collection: The questionnaire collected data on demographics (sex, age, and residence), primary activity setting (indoor/outdoor), Fitzpatrick skin phototypes (I–VI), history of sunburn episodes (<2, 2–5, 6–10, >10), number of nevi (0, <20, >20), smoking status, alcohol consumption, age at melanoma diagnosis, and melanoma-specific characteristics, including the Breslow index, histological subtype (superficial spreading melanoma [SSM], lentigo maligna melanoma [LMM], nodular melanoma [NM], acral lentiginous melanoma [ALM], and other types), tumor localization, family history of melanoma and the number of melanomas (single/multiple). Multiple melanomas are defined as both multiple synchronous primaries and second primary melanomas.

**Statistical Analysis**: Statistical analysis was conducted using MedCalc^®^ Statistical Software version 23.0.2 (MedCalc Software Ltd., Ostend, Belgium; https://www.medcalc.org; 2024). Nominal variables were presented as frequencies and percentages. Quantitative variables were expressed as medians and 25th–75th percentiles, as the distribution was non-normal (Shapiro–Wilk test). The Chi-square test was used to evaluate differences between nominal variables. Differences in quantitative data between groups were assessed using the Mann–Whitney or Kruskal–Wallis tests. A *p*-value of <0.01 was considered statistically significant.

## 3. Results

This study included 172 patients diagnosed with cutaneous melanoma (CM), of whom 57.6% were female. The clinical and demographic characteristics of the study population are presented in Table 1. The median age of the participants was 59 years (46.25; 70.00), with a median age at diagnosis of 54 years (44.00; 65.00). Among the participants, 119 (69.2%) resided in urban areas, and 103 (59.9%) primarily engaged in indoor activities.

Additionally, 81.4% of the patients were non-smokers, and 34.3% reported no alcohol consumption. Multiple melanomas were observed in 9 patients (5.2%), while 11 patients (6.4%) had a positive family history of melanoma.

The median Breslow index (BI) was 1.5 mm (0.70; 3.00).

The tumor characteristics and distribution by demographics are presented in Table 2. Although the median BI increased with advancing age, this trend did not reach statistical significance (*p* = 0.176). A notable 39% of patients had a high Breslow index (>2 mm), with a female predominance observed in all age groups.

Superficial spreading melanoma (SSM) was the most frequent histological subtype in both males (54, 74.0%) and females (80, 80.8%). Nodular melanoma (NM) was more prevalent in males (16, 21.9%) compared to females (12, 12.1%); however, the difference was not statistically significant (*p* = 0.309).

Males exhibited a significantly higher mean BI (1.7 mm) compared to females. Additionally, a higher BI was found in patients with NM, acral lentiginous melanoma (ALM), and non-smoker status (Table 3).

Significant differences in the median age at diagnosis were observed based on patient characteristics such as phototype, primary activity setting, presence of nevi, and smoking status (Table 3).

Regarding tumor localization, the limbs were the most frequent site in females (48.5%), whereas the trunk was predominant in males (67.1%), with this difference reaching statistical significance (*p* = 0.002). Males reported a higher prevalence of more than five sunburns (86.3%) compared to females (77.8%), although the difference was not statistically significant (*p* = 0.486). However, patients residing in rural areas reported a significantly higher proportion of more than ten sunburns in their history compared to urban residents (69.8% vs. 59.7%, *p* = 0.008).

No statistically significant association was found between the primary activity setting (indoor/outdoor) and histological subtype (*p* = 0.247). Similarly, multiple melanomas were not significantly associated with a history of sunburns, presence of nevi, smoking status, alcohol consumption, family history of melanoma, histological subtype, tumor localization, or phototype (Table 4).

## 4. Discussion

We present the findings on the characteristics and risk factors of cutaneous melanoma (CM) in the northwestern region of Romania. Our study revealed a predominance of female patients and a high proportion (39%) of advanced-stage melanoma, with a Breslow index (BI) >2 mm.

A female predominance was observed across all age groups in our cohort. Similarly, data from the Spanish National Cutaneous Melanoma Registry indicated a 13% higher frequency of tumors in women compared to men [21]. In France, slightly higher incidence rates in women (6.7/100,000) than in men (5.9/100,000) have also been reported [22]. These findings contrast with the epidemiology in the United States, where higher incidence rates are observed in men [23]. The observed female predominance could be attributed to greater health awareness among women, leading to earlier consultations with healthcare providers. Another hypothesis involves gender differences in sun exposure behaviors during adolescence and early adulthood, which might influence the higher rates of CM in younger women [24].

The median age of the participants in our study was 59 years, with the median age at diagnosis being 54 years. In comparison, Spanish studies reported a mean diagnosis age of 57 years, while the median age at diagnosis in Serbia was 57 years, with most cases in the 41–60-year age group [21,25,26]. In a study from Valencia, 10% of patients were under the age of 40, a proportion comparable to our finding of 9.3% [27]. Furthermore, 47.09% of our patients were older than 60 years, consistent with findings in Serbia where 52.6% were diagnosed over 60 years [26].

Lighter phototypes have been identified as significant risk factors for CM [28]. A Scottish study of 371 melanoma patients reported a lower risk in individuals with better tanning ability [29]. In Romania, phototypes II and III are predominant, which aligns with the findings of our study, where 44.8% of patients had phototype II and 46.5% had phototype III [30]. Interestingly, darker phototypes (III, IV) in our cohort were more frequent at a younger age at melanoma diagnosis.

Ultraviolet radiation (UVR) exposure remains the primary environmental risk factor for CM, although the effect is modulated by genetic factors, melanin content, and UV wavelength [31]. UVR exposure induces melanocyte transformation by causing DNA damage in the skin [32]. Furthermore, the timing and frequency of sun exposure have been strongly linked to an increased risk of CM in several studies [33,34]. A history of sunburn, particularly during childhood, has been more frequently found in patients with CM [1]. Several studies have shown a proportional increase in melanoma risk with the number of sunburns experienced [35,36]. In our study, 62.8% of patients reported experiencing more than ten sunburns. A Norwegian study emphasized that childhood is a critical period during which sunburn significantly increases the risk of CM, underscoring the importance of photoprotection measures in children [37].

A Romanian study indicated a higher risk of melanoma among individuals with outdoor or partially outdoor professions [35]. However, in our study, the majority of patients resided in urban areas and primarily engaged in indoor activities. Additionally, an indoor primary activity setting was significantly associated with a younger age at diagnosis (*p* < 0.001). Similarly, a study in the Czech Republic found that individuals working indoors but participating in outdoor hobbies were more likely to develop CM than those exposed to sun radiation, primarily during active sunbathing [38]. This could be attributed to a common lifestyle in both countries, such as gardening or spending time at rural cottages, combined with inadequate sun protection. This lifestyle results in unplanned sun exposure, often during the strongest midday sunlight (11 AM–3 PM).

In our study, patients living in rural areas reported a significantly higher proportion of more than ten sunburns in their history compared to urban residents (69.8% vs. 59.7%, *p* = 0.008). Additionally, males reported a higher prevalence of more than five sunburns (86.3%) compared to females (77.8%), although this difference was not statistically significant. Romania’s substantial rural population (46%) and the limited availability of physicians in these areas may partly explain the reported lower melanoma incidence rates in rural regions [39]. However, some Eastern European countries have reported a higher prevalence of thick melanomas in rural areas, suggesting that melanomas in these regions may remain underdiagnosed or be identified at more advanced stages [3,4].

A high nevus count has been identified as one of the most critical host risk factors for melanoma across all age groups, as reported in a study by Palve et al. [8]. Similarly, research conducted in the Florence area of central Italy demonstrated that the number of common melanocytic nevi was the strongest risk factor for cutaneous malignant melanoma, with no significant association observed with pigmentary traits [40]. In Sweden, a study found a strong correlation between a high number of nevi and an increased risk of melanoma, particularly in younger women (<40 years) [41]. Conversely, Tsao et al. concluded that moles persisting into old age present a higher risk for malignancy [14].

In our study, the presence of nevi was significantly linked to a younger age of melanoma diagnosis (*p* = 0.001). Although the mean Breslow Index (BI) was lower in patients with more than 20 nevi (mean BI = 1.2 mm) compared to those without nevi (mean BI = 1.8 mm), this difference did not reach statistical significance. Similar studies have shown that higher nevus counts correlate with earlier melanoma detection and slower tumor growth [42,43]. It is plausible that individuals with a higher number of melanocytic nevi are more likely to practice regular skin self-examinations, seek medical advice, and undergo mole removal procedures. These behaviors may contribute to the earlier detection and diagnosis of melanoma in this population [10,44]. Furthermore, immunosuppression and indoor tanning are recognized as significant risk factors for melanoma; however, these risk factors were not assessed in the questionnaire. [12]

Superficial spreading melanoma (SSM) was the most frequently observed histological subtype in both sexes (77.9%), followed by nodular melanoma (NM), which was more prevalent among males (21.9%). These findings align with a study conducted in Serbia, where SSM was the most common subtype (50–66%), followed by NM (23.5–50%) [26]. The majority of NM cases were observed in males older than 50 years, although this difference was not statistically significant [26]. Similarly, a Spanish study reported that SSM was the most frequently diagnosed subtype in both sexes, followed by NM [21]. SSM appears to be more commonly associated with individuals of lighter phototypes (I–III). For instance, a Brazilian study from the south and southwest regions, where melanoma patients are typically characterized as lighter phototypes (I–III), found that SSM was the predominant histological subtype. However, in northern Brazil, acral lentiginous melanoma (ALM) was more common, with the majority of patients being non-white [45].

Cutaneous melanoma can develop anywhere on the body but most frequently occurs in areas with chronic sun exposure [46]. In our study, the limbs were the most affected site in females (48.5%), whereas the trunk was predominant in males (67.1%). These findings are consistent with studies conducted in Spain, France, Hungary, and Serbia, which also reported the trunk as the most frequent site, followed by the legs. In these studies, the legs were more commonly affected in women, while the trunk was more commonly affected in men, potentially reflecting differences in sun exposure patterns between sexes [21,22,47,48]. However, a study from the United States reported that the trunk had become the most frequent site of melanoma in both men and women [49].

Interestingly, inverse associations between smoking and melanoma incidence were reported in three meta-analyses investigating the effects of smoking on melanoma risk [50,51,52]. In our study, smoking status was found often in patients with a younger age at diagnosis and a lower Breslow Index (*p* = 0.001). Regarding alcohol consumption, we did not observe any association between alcohol use and the Breslow Index, age of diagnosis, or the risk of developing multiple melanomas.

A family history of melanoma has been linked to disease characteristics. Palve et al. found that younger adults were more likely to report a family history of melanoma compared to middle-aged patients [8]. In contrast, other studies indicated that patients over 40 years of age were more likely to report a positive family history. Li et al. reported that patients with a positive family history were more likely to develop thicker melanomas [53]. However, other studies, consistent with our findings, found no significant differences in Breslow thickness between familial and sporadic melanomas [54].

Multiple primary melanomas (MPM) were observed in 5.2% of patients in our study, consistent with a Swedish cohort where 4% of 206 women had MPM [41]. Lentigo maligna and melanomas on the head and neck were associated with a higher risk of subsequent melanomas compared to SSM [17,55]. Lighter phototypes, male sex, older age, family history of melanoma, and high nevus counts were also significant risk factors [17,55,56]. However, sun exposure and hair color, despite their association with first primary melanomas, were not linked to increased MPM risk [56,57]. Our study found no significant associations between MPM and sunburns, nevi presence, smoking, alcohol use, family history, tumor localization, or phototype, possibly due to the small sample size.

In our study, the median Breslow Index (BI) was 1.5 mm, with 39% of patients presenting with a BI > 2 mm. While the median BI increased with age, this trend was not statistically significant (*p* = 0.176). Higher BI was linked to nodular melanoma (NM) and acral lentiginous melanoma (ALM). In Hungary, the mean BI significantly decreased from 2.2 mm to 1.6 mm between 1998 and 2008, whereas Serbia reported a median BI of 3 mm, with 31.95% of patients having tumors >4 mm [48,58]. Similarly to our findings, 39.59% of melanomas in Serbia exhibited a Breslow thickness of <1 mm, while in our study, 37.2% of patients had a BI <1 mm [48]. In Sweden, however, the median Breslow thickness for the first primary invasive cutaneous melanoma (CM) was notably lower, at 0.75 mm [41]. Also, age and male sex were associated with thicker tumors (>2 mm), similar to findings from Hungary and Australia, where older patients had increased Breslow thickness [43,47].

The later diagnosis of melanoma in Eastern Europe compared to other parts of Europe may be attributed to differences in general education levels, limited government funding for health education and screening programs, and a lack of public awareness. While skin cancer prevention campaigns were introduced in Western Europe in the 1980s, many countries in Central and Eastern Europe (CEE) have only recently launched initiatives such as Euromelanoma [59,60].

Patients often seek medical attention late, typically when skin lesions become alarming, often accompanied by symptoms such as ulceration or bleeding, which are indicative of advanced disease stages [61]. Given the poor prognosis and lack of highly effective treatment options for advanced melanoma, primary and secondary prevention strategies remain the most effective approaches for reducing melanoma-related mortality [62].

This study is the first to evaluate the associated risk factors of cutaneous melanoma in the Romanian population. Our findings align with prior international studies while offering new data specific to the Romanian population. The results may help guide strategies for more targeted patient education and screening initiatives.

Nonetheless, our study has several limitations. First, the sample size was relatively limited, consisting of an analysis of the prior history of malignant disease in 172 patients, including a notably small subset diagnosed with multiple melanomas. While we identified trends in risk factors and tumor characteristics, these findings require validation in larger, multicenter cohorts. Second, the study was restricted to the northwestern region of Romania, and the findings may not yet be generalizable to the entire country. Additionally, there is potential for recall bias, as skin cancer patients may overreport or underreport their history of sunburns, leading to inaccurate estimations of the true associations.

## 5. Conclusions

In many European countries, melanoma cases are reported to national or regional cancer registries. However, several Eastern European countries lack high-quality centralized cancer registries, which may lead to an underestimation of melanoma incidence and mortality rates. This study underscores the importance of establishing and maintaining a dedicated population-based registry for melanoma patients, emphasizing its critical role in accurate data collection and surveillance. The preliminary findings of this study highlight the pressing need for comprehensive public health campaigns that focus on both primary and secondary melanoma prevention. Future studies with larger, representative samples are needed to confirm these trends and guide targeted interventions.

## Figures and Tables

**Table 1:** Clinical and demographic characteristics of the study population.

	N (%)
Median age of the population (years)	59
Median age of diagnosis (years)	54
Sex	
Female	99 (57.6%)
Male	73 (42.4%)
Residence	
Urban	119 (69.2%)
Rural	53 (30.8%)
Primary activity setting	
Indoor	103 (59.9%)
Outdoor	69 (40.1%)
Fitzpatrick phototype	
I	3 (1.7%)
II	77 (44.8%)
III	80 (46.5%)
IV	12 (7.0%)
Smoking status	
Smoker	32 (18.7%)
Non-smoker	140 (81.3%)
Alcohol consumption	
Yes	113 (65,7%)
No	59 (34.3%)
Family history for melanoma	
Positive	11 (6.4%)
Negative	161 (93.6%)

**Table 2:** Tumor characteristics and distribution by demographics.

Tumor Localization		
Hands/feet	5 (2.9%)	
Head and neck	14 (8.1%)	
Limbs	63 (36.6%)	
Trunck	90 (52.3%)	
Histological subtype		
ALM	4 (2.3%)	
LM	2 (1.2%)	
NM	28 (16.3%)	
SSM	134 (77.9%)	
Others	4 (2.3%)	
Age group (years)	Breslow index (mm)	*p*-value
<40	0.8 (0.52; 1.57)	*p* = 0.176
40–60	1.4 (0.60; 3.00)	
>60	1.8 (0.70; 3.00)	
Breslow index (mm)	N (%)	
<1	64 (37.2%)	
1–2	41 (23.8%)	
>2	67 (39.0%)	

**Table 3:** Risk factor analysis.

History of Sunburns				
<2		13 (7.6%)		
2–5		19 (11.0%)		
6–10		32 (18.6%)		
>10		108 (62.8%)		
Number of nevi				
0		13 (7.6%)		
<20		81 (47.1%)		
>20		78 (45.3%)		
	BI (mm)	*p*-value	Mean age of diagnosis (years)	*p*-value
Tumor localization		*p* = 0.414		
Hands/feet	3.0 (1.25; 4.00)			
Head and neck	1.5 (0.77; 2.95)			
Limbs	1.2 (0.56; 3.00)			
Trunck	1.5 (0,70; 3.00)			
Residence		*p* = 0.361		
Urban	1.4 (0.60; 3.00)		54 (41.00; 66.00)	*p* = 0.413
Rural	1.8 (0.70; 3.00)		55 (46.00; 66.00)	
Sex		*p* = 0.008		
Female	1.1 (0.60; 2.50)		54 (44.00; 64.00)	*p* = 0.541
Male	1.7 (0.85; 4.00)		54 (43.00; 67.00)	
Phototype		*p* = 0.082		
Lighter (I/II)	1.6 (0.80; 3.00)		57 (46.50; 66.00)	*p* = 0.015
Darker (III/IV)	1.15 (0.51; 2.77)		49 (40.00; 63.00)	
History of sunburns		*p* = 0.453		
<5	1.45 (0.46; 3.60)			
>5	1.5 (0.70; 3.00)			
Histological subtype		*p* = 0.000		*p* = 0.156
ALM	3.5 (1.27; 4.00)		61 (47.50; 74.50)	
LM	0.65 (0.30;-)		58 (52.00; -)	
NM	3.25 (2.50; 4.87)		58 (49.25; 65.25)	
SSM	1.10 (0.54; 2.20)		53.5(42.75; 65.00)	
Others	1.15 (0.70; 2.57)		32 (26.50; 59.25)	
Number of nevi		*p* = 0.817		
None	1.8 (0.70; 3.25)			
<20	1.5 (0.60; 3.00)			
>20	1.2 (0.70; 3.00)			
Smoking status		*p* = 0.001		*p* = 0.001
Non-smoker	1.5 (0.70; 3.00)		57 (45.00; 66.00)	
Smoker	1.05 (0.52; 2.05)		44 (33.00; 57.75)	
Alcohol consumption		*p* = 0.684		*p* = 0.131
Yes	1.5 (0.70; 2.90)		52 (41.50; 63.00)	
No	1.5 (0.70; 3.20)		58 (48.00; 66.00)	
Family history for melanoma		*p* = 0.691		*p* = 0.915
Positive	1.5 (0.60; 3.00)		49 (45.00; 63.00)	
Negative	1.4 (0.70; 3.00)		54 (43.00; 65.00)	
Number of melanomas		*p* = 0.794		
Single	1.5 (0.70; 3.00)			
Multiple	1.5 (0.90; 4.90)			
Primary activity setting				*p* = 0.000
Indoor			49 (40.00; 61.00)	
Outdoor			60 (49.00; 68.00)	
Nevi				*p* = 0.001
No			58.5(47.00; 68.00)	
Yes			49 (39.00; 62.25)	

**Table 4:** Multiple melanomas and related characteristics.

	Patients with Multiple Melaomas (%)	*p*-Value
History of sunburns		*p* = 1.000
<5	1	
>5	8	
Nevi		*p* = 1.000
No	5	
Yes	4	
Smoking status		*p* = 0.674
Non-smoker	7	
Smoker	2	
Alcohol consumption		*p* = 1.000
Yes	6	
No	3	
Family history for melanoma		*p* = 0. 456
Positive	1	
Negative	8	
Histological subtype		*p* = 0.931
ALM	0	
LM	0	
NM	1	
SSM	8	
Others	0	
Tumor localization		*p* = 0.935
Hands/feet	0	
Head and neck	1	
Limbs	3	
Trunck	5	
Phototype		*p* = 0.306
Lighter (I/II)	6	
Darker (III/IV)	3	

## Data Availability

All data are available upon request from the correspondence author.
